# Evaluation of the Relationship Between Serum miR-200b-3p and miR-214-3p Expression Levels with Soluble ACE2 and TMPRSS2 in COVID-19 Patients

**DOI:** 10.5812/ijpr-137832

**Published:** 2023-10-11

**Authors:** Faezeh Mortazavi, Mohsen Soltanshahi, Gholamhossein Tamaddon

**Affiliations:** 1 Diagnostic Laboratory Sciences and Technology Research Center, School of Paramedical Sciences, Shiraz University of Medical Sciences, Shiraz, Iran; 2 Department of Immunology, Medical School, Shahid Beheshti University of Medical Sciences, Tehran, Iran

**Keywords:** ACE2, COVID-19, TMPRSS2, miR-200b-3p, miR-214-3p

## Abstract

**Background:**

The emergence and rapid global spread of the coronavirus disease 2019 (COVID-19) has presented a significant global health challenge. Severe acute respiratory syndrome coronavirus 2 (SARS-CoV-2) infects human host cells through the interaction of angiotensin-converting enzyme 2 (ACE2) and transmembrane serine protease 2 (TMPRSS2), which serve as main regulators for viral entry. Specifically, ACE2 and TMPRSS2 genes are influenced by two microRNAs: miR-200b-3p and miR-214-3p, respectively. The objective of this study was to explore the association between the serum levels of miR-200b-3p and miR-214-3p and the presence of circulating ACE2 and TMPRSS2 in severe and non-severe cases of COVID-19.

**Objectives:**

This study sought to examine the potential utility of microRNAs as biomarkers for assessing disease severity and progression. Additionally, the study aimed to elucidate the interplay between microRNAs and the ACE2 and TMPRSS2 proteins, which play crucial roles in facilitating SARS-CoV-2 viral entry and infection.

**Methods:**

This practical-foundational study involved the collection of samples from 61 hospitalized patients with confirmed COVID-19 and 31 healthy individuals. Subsequently, the enzyme-linked immunosorbent assay (ELISA) technique was utilized to measure the concentrations of ACE2 and TMPRSS2 in the blood samples. Additionally, the expression levels of serum miR-200b-3p and miR-214-3p were analyzed using real-time polymerase chain reaction (PCR). The statistical analysis of the data was conducted using GraphPad Prism software (version 8.02) and SPSS software (version 19.0), ensuring the accurate interpretation of results.

**Results:**

The findings revealed significant increases in the peripheral blood concentrations of ACE2 and TMPRSS2 in patients with non-severe COVID-19, compared to healthy individuals (P < 0.001 and P < 0.01, respectively). Similarly, patients with severe COVID-19 exhibited higher serum levels of ACE2 and TMPRSS2 than healthy subjects (P < 0.0001). Additionally, the serum levels of miR-200b-3p and miR-214-3p were decreased in both non-severe and severe COVID-19 patients, compared to healthy individuals (P < 0.01 and P < 0.0001, respectively). Moreover, a decrease in the serum levels of both miR-200b-3p and miR-214-3p was observed in patients with severe COVID-19, compared to those with non-severe cases (P < 0.001). Furthermore, this study identified a negative correlation between miR-200b-3p and ACE2 serum levels and between miR-214-3p and TMPRSS2 peripheral blood levels.

**Conclusions:**

The above-mentioned findings suggest that miR-200b-3p and miR-214-3p might be potential biomarkers for disease severity and prognosis in COVID-19 patients.

## 1. Background

The emergence of the coronavirus disease 2019 (COVID-19) in late December 2019, originating in the city of Wuhan, Hubei province, China, has resulted in a detrimental global pandemic, affecting over 200 countries worldwide (1-3). This infectious respiratory disease is caused by a novel coronavirus known as severe acute respiratory syndrome coronavirus 2 (SARS-CoV-2), which has been designated as the COVID-19 virus (4, 5). The disease caused by this virus manifests as a spectrum, ranging from subclinical infections to severe clinical complications, such as acute respiratory distress syndrome (ARDS), systemic hyperinflammatory response, and multiple organ failure. Notably, critical lung failure is the primary cause of morbidity and mortality associated with COVID-19 (1, 2, 5, 6). Several risk factors for fatal infection, including advanced age, male gender, smoking, pre-existing hypertension, and underlying heart, lung, and kidney diseases, have been identified (2, 5).

Two critical enzymes, angiotensin-converting enzyme 2 (ACE2) and transmembrane serine protease 2 (TMPRSS2), play crucial roles as the entry receptors for SARS-CoV-2 spike protein (1, 2, 6). Angiotensin-converting enzyme 2 is a cell membrane-attached enzyme that can be found in different cells and tissues of the body, including the digestive system, kidney, central nervous system, nasal and oral mucosa, and cardiovascular system. It is mainly expressed in type I and type II alveolar epithelial cells in the lungs (2, 7-9). In the renin-angiotensin-aldosterone system (RAAS), ACE2 cleaves angiotensin-1 and angiotensin-2 into Ang1-9 and Ang1-7, respectively, which helps maintain blood pressure regulation and electrolyte balance in the human body (1, 2, 5).

Studies have shown that the circulating level of ACE2 is directly related to the severity of respiratory infections caused by COVID-19 (4, 10, 11). Angiotensin-converting enzyme 2 is primarily expressed on the apical surface of epithelial cells in the airways, making it a target for the SARS-CoV-2 virus. When cells infected with the virus lyse, ACE2 is released into the bloodstream. This can lead to increased levels of ACE2 in the serum, which has been linked to more severe COVID-19 symptoms (1, 2, 4, 5). Alongside its membrane-bound form, increased circulating levels of ACE2 in the body indicate enhanced cell lysis due to virus infection, leading to proteolytic shedding. Consequently, soluble forms of this protein can be detected in the serum (4, 12). The elevated plasma concentration of ACE2 has been previously proposed as a biomarker for major cardiovascular events and inflammatory conditions (2, 4, 8). Recent studies have shown a significant elevation in plasma ACE2 levels in obese patients with COVID-19 and in individuals who smoke or have diabetes (2, 13-15).

Transmembrane serine protease 2 is a transmembrane serine protease found prominently in epithelial cells in various human organs, including the pulmonary and gastrointestinal tracts and the prostate (4, 16, 17). It is responsible for cleaving the SARS-CoV-2 spike protein into two subunits: The N-terminal S1 subunit and the C-terminal S2 subunit. This cleavage enables direct fusion between the host cell’s S2 subunit and cellular membranes, thereby facilitating viral entry into the target cells (3, 16-18). Additionally, TMPRSS2 has been found to support the entry of other viruses, including the influenza virus and human coronaviruses, such as human coronavirus 229E (HCoV-229E), Middle East respiratory syndrome-related coronavirus (MERS-CoV), severe acute respiratory syndrome coronavirus 2 (SARS-CoV), and most recently SARS-CoV-2, by cleaving viral envelope glycoproteins (4, 16, 17). Nevertheless, there is limited research available on the normal range of circulating TMPRSS2 levels in patients with COVID-19. In this context, a study has reported elevated levels of TMPRSS2 in the circulation during the early stages of the disease (4, 16-18).

MicroRNAs (miRNAs) are small, single-stranded, non-coding ribonucleic acids (RNAs) composed of 18 - 24 nucleotides. They play a negative regulatory role in gene expression at the translation level by binding complementarily to their target mRNAs, leading to mRNA degradation and/or translation repression (1, 6, 19). Aside from their involvement in physiological processes, such as proliferation, differentiation, and apoptosis, aberrant miRNA expression is associated with various pathological conditions (6, 20). miRNAs are found in different body fluids, including saliva, semen, urine, and blood. The screening of circulating miRNA profiles is used as a clinical biomarker to predict disease progression, diagnosis, and response to treatment (20, 21). Recent studies have suggested that circulating miRNAs hold promise as biomarkers for identifying COVID-19 cases (1, 3, 19, 20). Among various miRNAs, miR-200b-3p and miR-214-3p specifically bind to the 3' untranslated regions (UTRs) of ACE2 and TMPRSS2 mRNAs, respectively, leading to the dysregulation of ACE2 and TMPRSS2 expression (19, 22-26).

## 2. Objectives

This study aimed to evaluate the potential of serum miR-200b-3p and miR-214-3p levels as serum biomarkers to assess COVID-19 severity and prognosis. To determine whether miRNAs can modulate serum ACE2 and TMPRSS2 levels, this study investigated the predictive value of serum miR-200b-3p and miR-214-3p levels in correlating with the concentrations of ACE2 and TMPRSS2 in peripheral blood, respectively, in patients with severe and non-severe COVID-19.

## 3. Methods

### 3.1. Study Subjects

A total of 61 adult COVID-19 patients (34 men and 27 women) admitted to Hazrat Ali Asghar Hospital in Shiraz, Iran, were recruited for this practical-foundational study between October and December 2021. Additionally, 31 healthy individuals (17 men and 14 women) were included as controls. Informed consent was obtained from all the participants. Upon admission, routine laboratory serum tests were conducted. The COVID-19 infection was confirmed using a combination of reverse-transcriptase polymerase chain reaction (RT-PCR) of a nasopharyngeal swab and chest computerized tomography (CT) scan, following the protocol set by Iran’s Health Ministry. Subsequently, the patients were divided into two groups: Severe (n = 22) and non-severe (n = 29) COVID-19 patients based on clinical and laboratory symptoms. The study design and protocol received approval from the Medical Research Ethics Committee of the Paramedical Faculty of Shiraz University of Medical Sciences (IR.SUMS.REC.1400.482).

### 3.2. Sample Acquisition and RNA Extraction

In this study 5 mL of whole blood samples were obtained from both COVID-19 cases and healthy controls. The samples were left at room temperature (RT) for 20 minutes and then centrifuged at 4000 × g for 5 minutes at room temperature (RI) to collect the serum. Serum cryotubes were stored at -80°C until further analysis.

Ribonucleic acid extraction was carried out using 200 μL of serum and the Qiagen miRNeasy Serum/Plasma Kit (Qiagen, Germany) following the manufacturer’s instructions. To validate the quality and quantity of the samples, gel electrophoresis, and a Nanodrop spectrophotometer were utilized. For all the measurements, the 260/280 ratio ranged between 1.8 and 2.1, and the 260/230 ratio ranged between 2.0 and 2.2. The extracted total RNA samples were stored at -80°C (4).

### 3.3. Quantification of Soluble ACE2 and TMPRSS2 Using ELISA

The enzyme-linked immunosorbent assay (ELISA) quantification was performed following the Sandwich-ELISA principle using the Elabscience ELISA kit (Houston, TX, USA). Micro-ELISA plates were coated with specific antibodies to measure the serum concentrations of ACE2 and TMPRSS2 in control, severe, and non-severe subpopulations. After washing, standards or samples were added, followed by the addition of biotinylated antibodies for ACE2 and TMPRSS2. Any free components were washed away, and the substrate solution was added. The enzyme-substrate reaction was terminated using a stop solution. The absorbance was measured at A450 nm/540 nm using a multi-scan plate reader (Synergy HI BioTek, Winooski, USA) (27). The optical density (OD) value obtained is proportional to the concentration of human ACE2. The detection range of the ACE2 kit was between 0.39 and 25 ng/mL (with an intra-assay coefficient of variation [CV] of 5.01 - 4.68), and the kit demonstrated a sensitivity of 0.023 ng/mL. Additionally, the detection range of the TMPRSS2 kit was between 0.16 and 10 ng/mL (with an intra-assay CV of 5.45 - 3.47), with a sensitivity of 0.10 ng/mL. The samples with insufficient serum or poor serum quality were excluded from the study.

### 3.4. Quantitative Real-Time PCR

For cDNA synthesis, the RT microRNA PCR kit obtained from Pars Gene Company (Tehran, Iran) was used, utilizing 2 μg of total RNA per sample. The RT enzyme was inactivated by heating the samples to 85°C for 1 minute. Subsequently, the samples were incubated at 42°C for 60 minutes. The miR-200b-3p and miR-214-3p quantitative real-time PCR (qPCR) assays were performed using an ABI 7500 Real-Time PCR System (Applied Biosystems, Foster City, CA, USA), SYBR Green Master Mix (Parsgenome, Tehran, Iran), and specific primers (Cat No. 00101007, Parsgenome Tehran, Iran). The sequence of miRNAs for the real-time PCR is provided in Table 1. To normalize the expression levels of the miRNAs, the U6 gene was used as the endogenous control. The real-time PCR protocol consisted of an initial denaturation step at 95°C for 15 minutes, followed by a 40-cycle amplification process, with each cycle including denaturation at 95°C for 30 seconds, annealing at 62°C for 30 seconds, and extension at 72°C for 30 seconds. 

**Table 1.** Sequence of miRNAs for Quantitative Real-Time Polymerase Chain Reaction (PCR)

miRNA	Sequence
**hsa-miR-200b-3p**	UAAUACUGCCUGGUAAUGAUGA
**hsa-miR-214-3p**	ACAGCAGGCACAGACAGGCAGU

The relative quantification of miRNAs was determined using the 2^−ΔΔCT^ method, normalized to the endogenous control (28). To predict target miRNA(s) and their corresponding targets, three different in silico prediction databases were utilized. Firstly, the miRanda miRNA target prediction algorithm (29) was employed to generate a list of miRNAs potentially binding to the 3' UTR of ACE2 and TMPRSS2 target mRNAs. Additionally, TargetScan (30) and miRTarBase (31) were utilized to determine the miRNA-mRNA target interactions by considering both predicted and validated miRNA-target gene interactions’ databases.

### 3.5. Statistical Analysis

All the experiments in this study were performed in triplicate, and the data were reported as mean ± standard error of the mean (SEM) values. The statistical analysis used GraphPad Prism software (version 8.02) and SPSS software (version 19.0). The two-way analysis of variance (ANOVA) statistical test was utilized to compare the data obtained from different groups, and the P-value < 0.05 was considered statistically significant.

## 4. Results

### 4.1. Characteristics of the Study Population

The present study included a total of 61 COVID-19 patients, with a mean age of 60.19 years (SEM = ± 2.15), and 31 healthy individuals, with a mean age of 55.4 (SEM = ± 1.78). The correlation between the serum levels of miR-200b-3p and miR-214-3p with ACE2 and TMPRSS2 was analyzed. The demographic and characteristic features of the enrolled COVID-19 patients are presented in Table 2. The most common symptoms observed upon hospital admission were fever (90%), fatigue (77%), and dry cough (70%), with diarrhea (14.7%), vomiting (11.4%), and hemoptysis (4.9%) being less common.

**Table 2.** Baseline Demographics and Clinical Characteristics of Coronavirus Disease 2019 (COVID-19) Patients with Comparison of Individuals in Severe and Non-severe Groups ^a^

Parameters	All Patients (N = 61)	Non-severe (N = 36)	Severe (N = 25)	P-Value ^b^
**Age, mean (SEM)**	60.19 (2.15)	59.63 (2.58)	63.90 (3.56)	0.2
**Male**	34 (55.7)	21 (58.3)	13 (52)	0.64
**Female**	27 (44.2)	15 (41.6)	12 (48)	0.5
**Fever**	55 (90.16)	30 (83.3)	25 (100)	0.02
**Fatigue**	47 (77.04)	26 (72.22)	21 (84)	0.2
**Dry cough**	43 (70.49)	21 (58.33)	22 (88)	0.01 ^c^
**Dyspnea**	34 (55.73)	15 (41.66)	19 (76)	0.006 ^c^
**Myalgia**	28 (35.90)	12 (33.33)	16 (64)	0.01 ^c^
**Sputum production**	9 (14.75)	8 (22.22)	1 (4)	0.05
**Anorexia**	30 (49.18)	13 (36.11)	17 (68)	0.01 ^c^
**Hemoptysis**	3 (4.91)	1 (2.77)	2 (8)	0.12
**Pharyngalgia**	15 (24.59)	9 (25)	6 (24)	0.7
**Nausea**	13 (21.31)	9 (25)	4 (16)	0.64
**Vomiting**	7 (11.47)	5 (13.88)	2 (8)	0.5
**Diarrhea**	9 (14.75)	6 (16.66)	3 (12)	0.6
**Dizziness**	21 (34.42)	11 (30.55)	10 (40)	0.42
**Headache**	20 (32.78)	13 (36.11)	7 (28)	0.5

Abbreviation: SEM, standard error of the mean.

^a^Values are expressed as No. (%) unless otherwise indicated.

^b^ The P-value represents differences between severe and non-severe patient groups.

^c^ A P-value less than 0.05 is considered statistically significant.

Based on routine laboratory testing and the results shown in Table 3, severe COVID-19 subjects exhibited significantly higher levels of neutrophils, lymphocytes, albumin, total bilirubin, alanine transaminase (ALT), aspartate aminotransferase (AST), blood urea nitrogen (BUN), lactate dehydrogenase (LDH), and C-reactive protein (CRP) than non-severe COVID-19 subjects. Moreover, the values of prothrombin time (PT), partial thromboplastin time (PTT), and erythrocyte sedimentation rate (ESR) were higher in severe COVID-19 subjects than in non-severe cases.

**Table 3.** Laboratory Findings of Coronavirus Disease 2019 (COVID-19) Patients ^a^

Variables	All Patients (N = 61)	Non-severe (N = 36)	Severe (N = 25)	P-Value ^b^
**WBC (10** ^**3**^ **/µL)**	7 (4.4 - 10.2)	6.6 (4.4 - 8.4)	7.6 (4.3 - 10.6)	0.2
**Neut (%)**	72.99 (47.3 - 87.16)	72.04 (46.9 - 83.5)	87.12 (83.3 - 92.7)	< 0.0001 ^c^
**Lymph (%)**	13.82 (5.7 - 18.85)	17.85 (8.3 - 27.4)	7.22 (3.3 - 10.3)	< 0.0001 ^c^
**PLT (10** ^**3**^ **/µL)**	199.51 (151 - 231)	209.16 (177 - 254)	183.72 (113 - 234)	0.05
**PT (s)**	12.86 (12 - 13.32)	12.41 (12 - 13)	13.58 (12 - 15)	0.0003 ^c^
**PTT (s)**	32.36 (26 - 39)	30.94 (25 - 35)	34.68 (29 - 42)	0.03 ^c^
**Hb (g/dL)**	11.1 (8.5 - 12.9)	11.49 (9.5 - 12.9)	10.5 (8.6 - 12.1)	0.05
**Albumin (g/dL)**	3.45 (3.1 - 3.8)	3.64 (3.1 - 3.9)	3.1 (2.4 - 3.6)	0.0006 ^c^
**Total bilirubin (mg/dL)**	1.04 (0.7 - 1.7)	0.83 (0.5 - 1.1)	1.39 (0.8 - 2.1)	< 0.0001 ^c^
**ALT (U/L)**	36 - 96 (27 - 56)	34 (25 - 43)	51.81 (31 - 68)	0.02 ^c^
**AST (U/L)**	45.5 (34 - 71)	42.58 (25 - 64)	59.86 (37 - 76)	0.03 ^c^
**BUN (mg/dL)**	38.18 (27 - 71)	34.75 (26 - 49)	51.36 (30.2 - 81)	0.007 ^c^
**Cr (mg/dL)**	1.3 (1 - 1.7)	1.47 (1 - 1.6)	1.75 (1.2 - 2.3)	0.08
**LDH (IU/L)**	502 (460 - 706)	513.25 (440 - 601)	633.4 (538 - 737)	0.005 ^c^
**CRP (mg/dL)**	70.51 (24.3 - 98.6)	49.69 (18 - 65)	104.57 (77 - 129.5)	< 0.0001 ^c^
**ESR (mm/h)**	50.60 (37 - 67)	45.33 (26 - 59)	59.22 (50 - 79.1)	0.005 ^c^
**Serum ferritin (ng/mL)**	786 (375.5 - 1161)	452 (303.5 - 720.6)	947 (841 - 1239)	0.06

Abbreviations: IQR, interquartile range; WBC, white blood cell; Neut, neutrophils; Lymph, lymphocytes; PLT, platelets; PT, prothrombin time; PTT, partial thromboplastin time; Hb, hemoglobin; ALT, alanine transaminase; AST, aspartate aminotransferase; BUN, blood urea nitrogen; Cr, creatinine; LDH, lactate dehydrogenase; CRP, C-reactive protein; ESR, erythrocyte sedimentation rate.

^a^ Values are expressed as median (IQR).

^b^ P-values were calculated to determine the statistical significance of differences between severe and non-severe patient groups.

^c^ P-values less than 0.05 were considered statistically significant.

### 4.2. Soluble ACE2 and TMPRSS2 Concentrations in COVID-19 and Healthy Subjects

**Figure 1.**
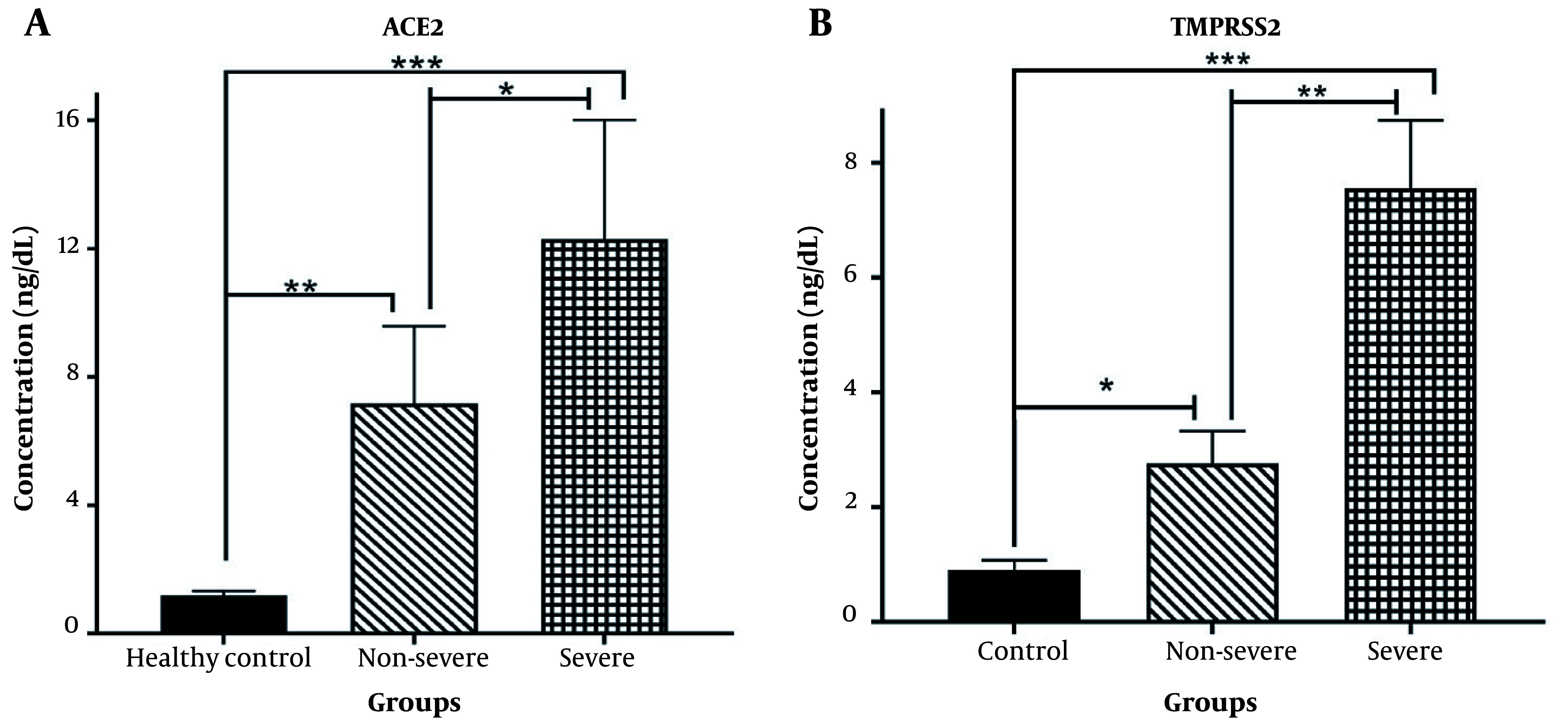
A, Soluble angiotensin-converting enzyme 2 (ACE); and B, transmembrane serine protease 2 (TMPRSS2) concentrations in healthy controls, non-severe, and severe disease groups (* P < 0.01, ** P < 0.001, *** P < 0.0001; P < 0.05 was considered significant).

The concentrations of soluble ACE2 and TMPRSS2 were examined in individuals infected with SARS-CoV-2 and healthy controls. As shown in Figure 1A, a significant increase in soluble ACE2 levels was observed in both non-severe and severe COVID-19 patients, compared to the healthy subjects (P < 0.001 and P < 0.0001, respectively). Moreover, the serum concentration of TMPRSS2 was significantly higher in patients with non-severe COVID-19 (P < 0.01) and severe COVID-19 (P < 0.0001) than in the control group. Additionally, the soluble serum concentrations of ACE2 and TMPRSS2 were elevated in severe COVID-19 patients, compared to those with non-severe COVID-19 (P < 0.01 and P < 0.001, respectively).

### 4.3. Validation of miR-200b-3p and miR-214-3p Expression Profiles in COVID-19 Patients and Healthy Subjects

**Figure 2.**
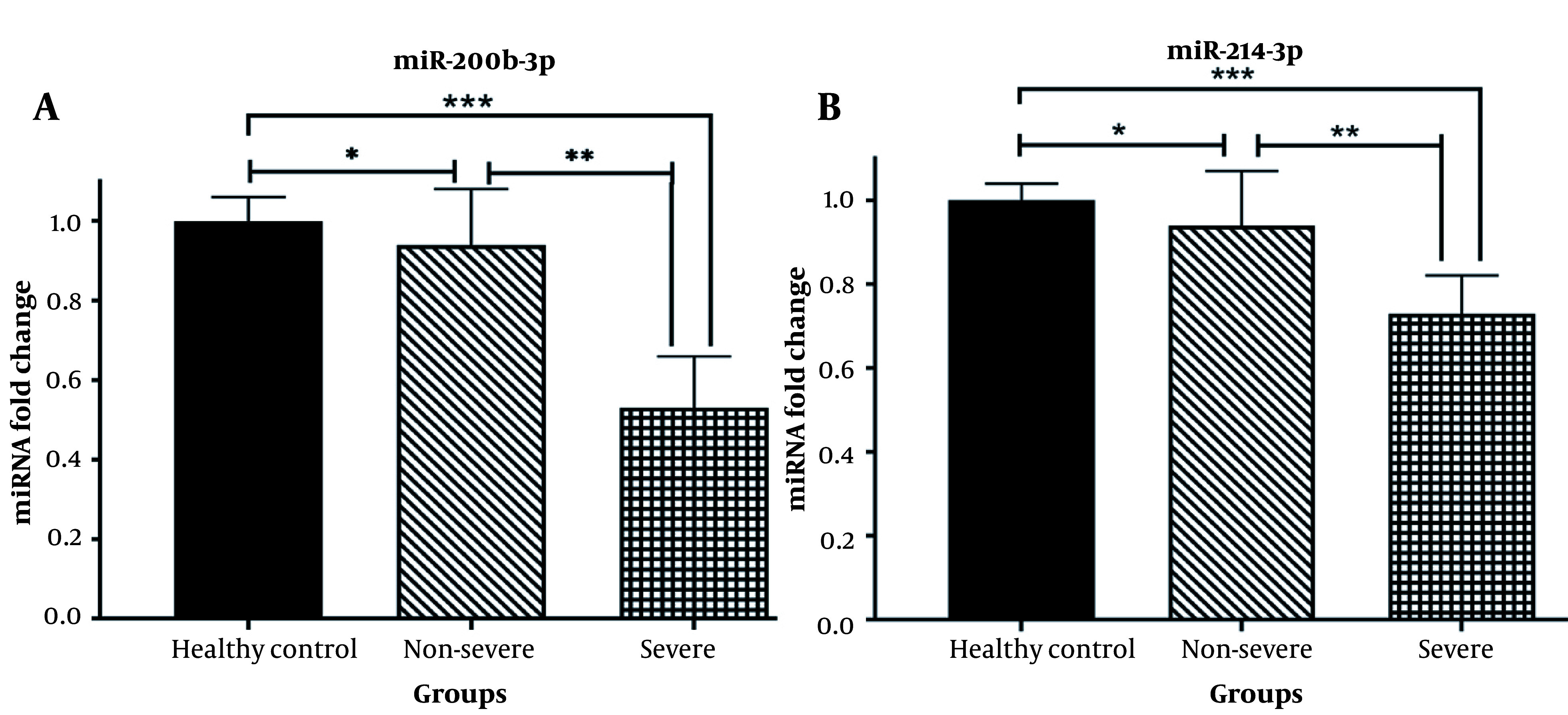
A, relative expression of serum miR-200b-3p; and B, miR-214-3p in healthy controls, non-severe, and severe disease groups (* P < 0.01, ** P < 0.001, *** P < 0.0001; P < 0.05 was considered significant).

An analysis was conducted to determine whether there are differences in the expression levels of serum miR-200b-3p and miR-214-3p, which regulate ACE2 and TMPRSS2, respectively, between patients’ blood samples and a healthy control group. Additionally, it was investigated whether there is a discrepancy in the serum levels of these two microRNAs between severe and non-severe patients. The results of real-time qPCR revealed a significant decrease in the expression levels of miR-200b-3p (Figure 2A) and miR-214-3p (Figure 2B) in the peripheral blood of patients with severe COVID-19, compared to the healthy population (P < 0.0001). Furthermore, the serum expression levels of miR-200b-3p (Figure 2A) and miR-214-3p (Figure 2B) were significantly downregulated in non-severe SARS-CoV-2 infected subjects compared to the healthy group (P < 0.01). Moreover, both miR-200b-3p and miR-214-3p exhibited decreased serum expression levels in severe patients compared to non-severe COVID-19 cases (P < 0.001).

### 4.4. Correlation Analysis of Serum miR-200b-3p and miR-214-3p Expression with Serum Concentrations of ACE2 and TMPRSS2 in Patients with Severe and Non-severe COVID-19

**Figure 3.**
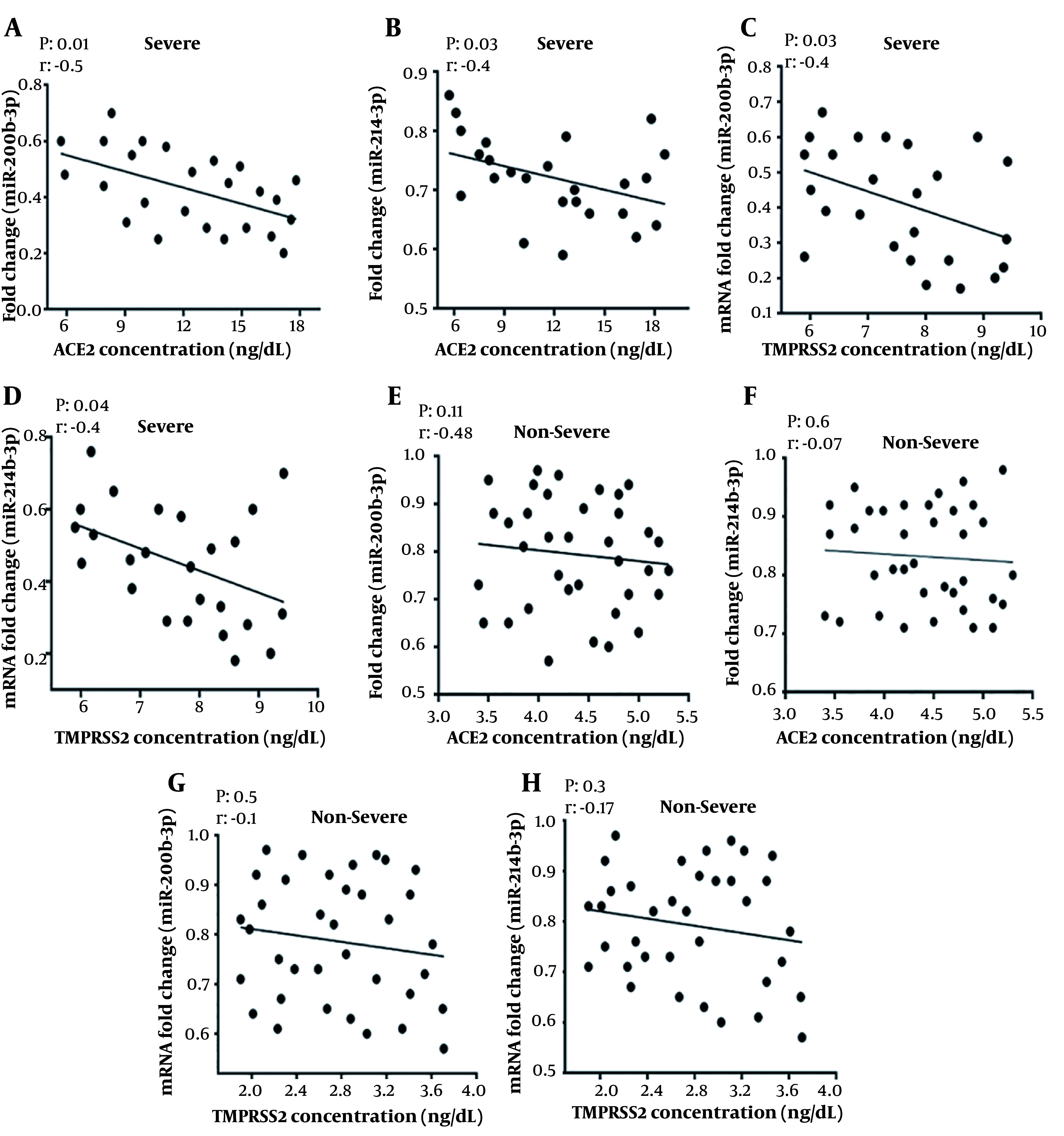
Correlation analysis of serum miR-200b-3p and miR-214-3p expression with soluble angiotensin-converting enzyme 2 (ACE2) and transmembrane serine protease 2 (TMPRSS2) concentrations in severe and non-severe coronavirus disease 2019 (COVID-19) patients; A, correlation analysis of soluble ACE2 and serum miR-200b-3p in severe COVID-19 patients; B, correlation analysis of ACE2 and miR-214-3p in severe patients; C, correlation analysis of TMPRSS2 and miR-200b-3p in severe patients; D, correlation analysis of TMPRSS2 and miR-214-3p in severe patients; E, correlation analysis of ACE2 and serum miR-200b-3p in non-severe COVID-19 patients; F, correlation analysis of ACE2 and miR-214-3p in non-severe patients; G, correlation analysis of TMPRSS2 and miR-200b-3p in non-severe patients; H, correlation analysis of TMPRSS2 and miR-214-3p in non-severe patients (The regression line is also illustrated. Pearson correlation [r] and P-value [P] are reported. A P-value < 0.05 was considered significant).

As shown in Figure 3, correlation analyses were conducted to assess the associations between the serum levels of miR-200b-3p, miR-214-3p, ACE2, and TMPRSS2 in patients with severe and non-severe COVID-19. The results showed that the concentration of ACE2 in severe COVID-19 patients was negatively correlated with the relative expression of miR-200b-3p (r = -0.5, P = 0.01) and miR-214-3p (r = -0.4, P = 0.03). Similarly, there was a significant negative correlation between the concentration of TMPRSS2 in severe COVID-19 patients and the relative expression of miR-200b-3p (r = -0.4, P = 0.03) and miR-214-3p (r = -0.4, P = 0.04). In contrast, no statistically significant correlation was observed between ACE2 concentration and relative expression levels of miR-200b-3p (r = -0.48, P = 0.11) or miR-214-3p (r = -0.07, P = 0.6) in non-severe COVID-19 patients. Likewise, there was no significant correlation between the concentration of TMPRSS2 and the relative expression of miR-200b-3p (r = -0.1, P = 0.5) or miR-214-3p (r = -0.17, P = 0.3) in non-severe COVID-19 patients. In conclusion, the correlations of ACE2 and TMPRSS2 levels with either miR-200b-3p or miR-214-3p were not statistically significant in non-severe COVID-19 patients.

## 5. Discussion

The identification of biological markers that possess significant diagnostic and prognostic value in predicting the progression of COVID-19 can have implications for the early detection, confirmation, and treatment of the disease (25). In this context, peripheral miRNAs have been introduced as minimally invasive biomarkers that hold potential in various aspects of COVID-19, including diagnosis and treatment. Recent advancements in understanding the molecular mechanisms underlying COVID-19 have highlighted the crucial roles played by miRNAs in the pathogenesis of this infection (7, 25). Therefore, miRNAs have emerged as promising candidates for the development of miRNA-based therapeutic interventions to enhance the clinical outcomes of COVID-19 (1, 25).

The present study specifically focused on identifying miR-200b-3p and miR-214-3p, which target ACE2 and TMPRSS2, respectively, as potential miRNA biomarkers for predicting the course of SARS-CoV-2 infection. To achieve this aim, in this study, the concentrations of soluble ACE2 and TMPRSS2 were measured. Subsequently, the serum levels of miR-200b-3p and miR-214-3p were assessed in severe and non-severe COVID-19 patients in comparison to healthy individuals.

This study hypothesized that SARS-CoV-2 infection would lead to the shedding of TMPRSS2 and ACE2 from cell membranes, resulting in increased plasma levels (8). This hypothesis is supported by the critical roles played by these two enzymes in the pathophysiology of the illness, particularly in the cell entry mechanisms of coronaviruses (4, 6). The processing of full-length ACE2 on the cell membrane by ADAM17 (i.e., a disintegrin and metalloprotease) produces soluble ACE2 (15, 32). The current study’s data revealed a significant increase in ACE2 and TMPRSS2 concentrations in the serum of both severe and non-severe COVID-19 patients compared to healthy subjects. However, this increase was more prominent in severe patients. Additionally, ACE2 and TMPRSS2 serum concentrations were higher in severe COVID-19 cases than in non-severe cases.

Hoffmann et al. indicated in a study that the elevated expression of ACE2 might render host cells more susceptible to SARS-CoV-2 infection (3). Furthermore, the experimental models of SARS-CoV infection have demonstrated that abundant ACE expression can contribute to increased viral dissemination and disease severity (15). Another study by Elemam et al. showed a significant enhancement of soluble ACE2 levels in COVID-19 patients compared to healthy individuals (33). Similarly, Fagyas et al. showed significantly elevated serum ACE2 levels in severely hospitalized COVID-19 patients compared to healthy individuals. However, they reported that increased ACE2 activity might not be considered a specific biomarker for COVID-19 (5). Serum ACE2 appears to be a nonspecific biomarker for systemic inflammation in COVID-19, as its levels increase during severe sepsis (5).

As miRNAs play a crucial role in regulating the immune response against infectious diseases and show early changes in disease onset even before pathogen detection, this study measured the serum levels of miR-200b-3p and miR-214-3p. These miRNAs were found to be significantly decreased in patients with both severe and non-severe COVID-19 compared to the healthy group. Specifically, miR-200b-3p and miR-214-3p serum levels exhibited a higher downregulation in severe COVID-19 patients than in non-severe cases. The aforementioned findings highlight the potential and clinical significance of miR-200b-3p and miR-214-3p as markers of COVID-19 severity.

Several miRNAs, including miR-200b-3p, miR-214-3p, miR-200c-3p, miR-125b, miR-18, miR-98-5p, and miR-27b, have been identified as targeting ACE2, TMPRSS2, or other bioactive molecules involved in the pathological processes of COVID-19 and other viral diseases. These miRNAs have shown both upregulation and downregulation in acute and critical patients, compared to healthy subjects (24, 25, 34-37). In other studies, the correlation between miR-200 and miR-24 with the regulation of ACE2 and furin, respectively, has been reported (38). Kaur et al. demonstrated that miR-214, miR-98, and miR-32 have a high affinity for binding sites on TMPRSS2 and effectively inhibit this protein (39).

Furthermore, the downregulation of miR-214 has been associated with higher TMPRSS2 expression and increased vulnerability to COVID-19 (40, 41). Chauhan et al. demonstrated that miR-200b-3p, miR-200c-3p, and miR-429 might inhibit ACE2 in their study (36). Therefore, measuring the serum concentrations of miRNAs involved in regulating ACE2 and TMPRSS2 as potential biomarkers during COVID-19 infection is a plausible approach. In the present study, the serum levels of miR-200b-3p and miR-214-3p were significantly decreased in patients with both severe and non-severe COVID-19 compared to the healthy group. Furthermore, miR-200b-3p and miR-214-3p serum levels were downregulated in patients with severe COVID-19, compared to non-severe COVID-19, with a higher downregulation in the severe COVID-19 patients. The aforementioned findings suggest the potential and clinical significance of miR-200b-3p and miR-214-3p as markers of severity in COVID-19.

In the current study, a correlation analysis was conducted between the concentrations of ACE2 and TMPRSS2 and the levels of circulatory miR-200b-3p and miR-214-3p in patients with severe and non-severe COVID-19. To the best of our knowledge, this is the first study to examine the profile of miR-200b-3p and miR-214-3p levels and investigate the relationship between serum concentrations of ACE2, TMPRSS2, and these two miRNAs in COVID-19. The findings revealed a negative correlation between the levels of miR-200b-3p, miR-214-3p, ACE2, and TMPRSS2 in the peripheral blood of severe COVID-19 patients. The aforementioned results suggest that reduced expression of miR-200b-3p and miR-214-3p in COVID-19 eliminates their inhibitory functions on ACE2 and TMPRSS2, respectively. Consequently, this leads to higher expression levels of these two enzymes, which can contribute to the deterioration of the disease course. The inverse relationship between ACE2 serum levels and miRNA levels has been previously reported. For instance, a study by Trojanowicz et al. demonstrated that increasing the blood miR-421-5p level results in a decrease in ACE2 levels (42, 43).

Similarly, Abdolahi et al. showed a significant decrease in the expression levels of miR-200c-3p and miR-421-5p upon admission. They concluded that modifying the expression of miR-200c-3p, miR-421-5p, and ACE concentrations might help reduce interleukin 6 (IL-6) levels and maintain hemostasis during COVID-19 infection (1). The results of the present study align with these previous findings. However, in the present study, there was no significant correlation between the levels of soluble ACE2 and TMPRSS2 and the serum levels of miR-200b-3p and miR-214-3p in non-severe patients.

Additionally, the current study also examined the demographic and clinical features of individuals with varying degrees of COVID-19. Among the clinical symptoms analyzed, dry cough, dyspnea, myalgia, and anorexia showed significant differences between patients with severe and non-severe COVID-19; however, the remaining symptoms did not reach significance. Consistent with the findings of previous clinical investigations (33), the current study detected higher levels of inflammatory markers in severe patients than in non-severe individuals using routine laboratory tests. However, further research is required to determine if miRNAs can serve as predictive markers in conjunction with inflammatory biomarkers in SARS-CoV-2-infected subjects.

There are certain limitations associated with this study. Firstly, the sample size was relatively small. Secondly, there was no direct comparison between severe and non-severe COVID-19 patients. Thirdly, non-COVID patients with respiratory distress were not included as a control group.

### 5.1. Conclusions

This study highlights the potential role of measuring serum levels of miR-200b-3p and miR-214-3p as early predictive indicators for forecasting the disease outcome of COVID-19 infection. The findings demonstrate a negative correlation between the downregulation of miR-200b-3p and miR-214-3p and the serum concentrations of ACE2 and TMPRSS2, respectively. Modulating these miRNAs could serve as therapeutic candidates to manipulate ACE2 and TMPRSS2 levels, thereby promoting treatment strategies for severe COVID-19. The aforementioned results have significant implications for further in vitro and in vivo investigations to elucidate the underlying mechanisms and monitor the serum levels of miR-200b-3p and miR-214-3p in correlation with peripheral blood concentrations of ACE2 and TMPRSS2 in post-COVID-19 survivors. This knowledge can contribute to the development of preventive and individualized therapeutic approaches in the near future.

## Data Availability

The dataset presented in the study is available on request from the corresponding author during submission or after publication.
